# Mouse Models of Human Claudin-Associated Disorders: Benefits and Limitations

**DOI:** 10.3390/ijms20215504

**Published:** 2019-11-05

**Authors:** Murat Seker, Cármen Fernández-Rodríguez, Luis Alfonso Martínez-Cruz, Dominik Müller

**Affiliations:** 1 Department of Pediatric Gastroenterology, Nephrology and Metabolism, Charité—Universitätsmedizin Berlin, Charité, 13353 Berlin, Germany; murat.seker@charite.de; 2 ClC BioGUNE, Bizkaia Science and Technology Park, 801A, 48160 Derio, Spain; cfernandez@cicbiogune.es (C.F.-R.); amartinez@cicbiogune.es (L.A.M.-C.)

**Keywords:** tight junction, claudin, mutations, kidney, liver, skin, human, mice, disease

## Abstract

In higher organisms, epithelia separate compartments in order to guarantee their proper function. Such structures are able to seal but also to allow substances to pass. Within the paracellular pathway, a supramolecular structure, the tight junction transport is largely controlled by the temporospatial regulation of its major protein family called claudins. Besides the fact that the expression of claudins has been identified in different forms of human diseases like cancer, clearly defined mutations in the corresponding claudin genes have been shown to cause distinct human disorders. Such disorders comprise the skin and its adjacent structures, liver, kidney, the inner ear, and the eye. From the phenotype analysis, it has also become clear that different claudins can cause a complex phenotype when expressed in different organs. To gain deeper insights into the physiology and pathophysiology of claudin-associated disorders, several mouse models have been generated. In order to model human disorders in detail, they have been designed either as full knockouts, knock-downs or knock-ins by a variety of techniques. Here, we review human disorders caused by CLDN mutations and their corresponding mouse models that have been generated thus far and assess their usefulness as a model for the corresponding human disorder.

## 1. Introduction

In higher organisms epithelia, endothelia and mesothelia are essential to separate different compartments in order to guarantee their proper function. Such structures can be found ubiquitously, like in skin which separates the body from the surrounding environment, the lung (air/blood) and the intestine (gut lumen/blood). Further, epithelial structures form the blood-brain-barrier [1] and are also essential for liver and kidney function [2]. Epithelia consist of monolayer or multilayer structures [3] but a common prerequisite for their function is polarization, i.e., a clear orientation of apical to basolateral requiring a well-defined maintenance molecular machinery [4]. Moreover, besides providing a tight barrier (e.g., within the bladder), epithelial structures are able to regulate coordinated transport across cellular barriers. Transport across epithelial barriers is provided in general via two systems, the paracellular and the transcellular pathway. Whereas the first provides the organism with a maximum of resorptive capacity by minimal energy expenditure, the latter aims at fine-tuning depending on the actual needs of the organism, regulated also by a subset of various local, regional and global mechanisms [5].

In more detail, the transcellular pathway consists of apical uptake, intracellular buffering, transport and basolateral extrusion mechanisms. This pathway is highly energy dependent and mostly driven by the basolateral Na^+^-K^+^-ATPase. One of the advantages of such a pathway is its controllability on several of the steps, e.g., by hormones (e.g., 1,25(OH)_2_D_3_ or Parathyroid Hormone (PTH)) and therefore also aims at fine-tuning to provide the organism according to its actual needs and also on middle and long term regulation [6]. Especially for these pathways, it has been shown that mutations in human gene cause distinct monogenetic disorders, like Bartter’s [7] and Gitelman’s Syndrome [8] with a clear phenotype-genotype correlation. In order to study such genetic effects, mouse models have been generated to study successfully the consequences of human mutations [9,10,11,12,13].

Considering the paracellular pathway, enormous progress has been achieved during the last two decades as it became clear that this pathway, although basically driven by a given electrochemical gradient only, is regulated by several structures and mechanisms. In regions where concentration gradients of solutes across the epithelial layer are high, like the proximal tubule of the kidney, paracellular pathways provide the organism with maximal reabsorption by minimal energy expenditure. More distantly, crosstalk takes place before the transcellular pathway provides the major mechanism. A key component of the paracellular pathway is a supramolecular structure, called tight junction (TJ). The TJ consists of several membrane-bound proteins and their intracellular adapter and scaffolding proteins [14]. The major proteins essential for the TJ are claudins (lat. *claudere*, i.e., to seal). The family of claudins is currently included 27 members in eukaryotes. The claudin proteins are membrane-bound and span four times the plasma membrane with an intracellular C- and N-terminal part. Each of the four provides a functional entity, i.e., either to act as pore-forming or as a sealing component (for a detailed review: [15]). Classification of claudins has been made based on different factors such as sequence similarity and functionality. In this review, we used the latter one which was based on sealing or pore-forming capabilities of claudins reviewed by Krause et al. [16].

In 1999, the group of Lifton showed that the disorder *Familial Hypercalciuria, Hypomagnesemia with Nephroclacinosis (FHHNC*) is caused by mutations in the *CLDN16* gene. This observation provided the first evidence that TJ disorders cause human disorders and diseases [17]. Since then, several other TJ disorders have been shown to cause, when mutated, (*CLDN1, CLDN2 CLDN9 CLDN10, CLDN14, CLDN16, CLDN19*), human disorders [18,19,20,21,22,23]. Furthermore, expression of claudins are influenced by many factors (e.g., smoking, diet changes, alcohol intake) [24,25,26] and numerous associations of disordered claudin expression and disease have been described. (for detail reviews see [27,28]).

To model such disorders, genetically modified mice have been generated. Initially, embryonic stem cell technology was used to create such models [29] and later, other approaches have been established successfully [30]. Besides global deletions, regional and local variations of murine gene expression have been established. Although not reported yet, based on current developments, it can be expected that CRISPR/Cas9 technology will take place as a standard procedure [31].

However, even though such models provide insights into physiology and pathophysiology and may open new avenues for the development of therapeutical interventions, they face limitations. Here, we provide an overview of current human claudin-associated disorders and their corresponding mouse models, their impact on physiology and pathophysiology.

## 2. Claudin Mutations Causing Human Disorders

### 2.1. Claudin 1

**Figure 1:**
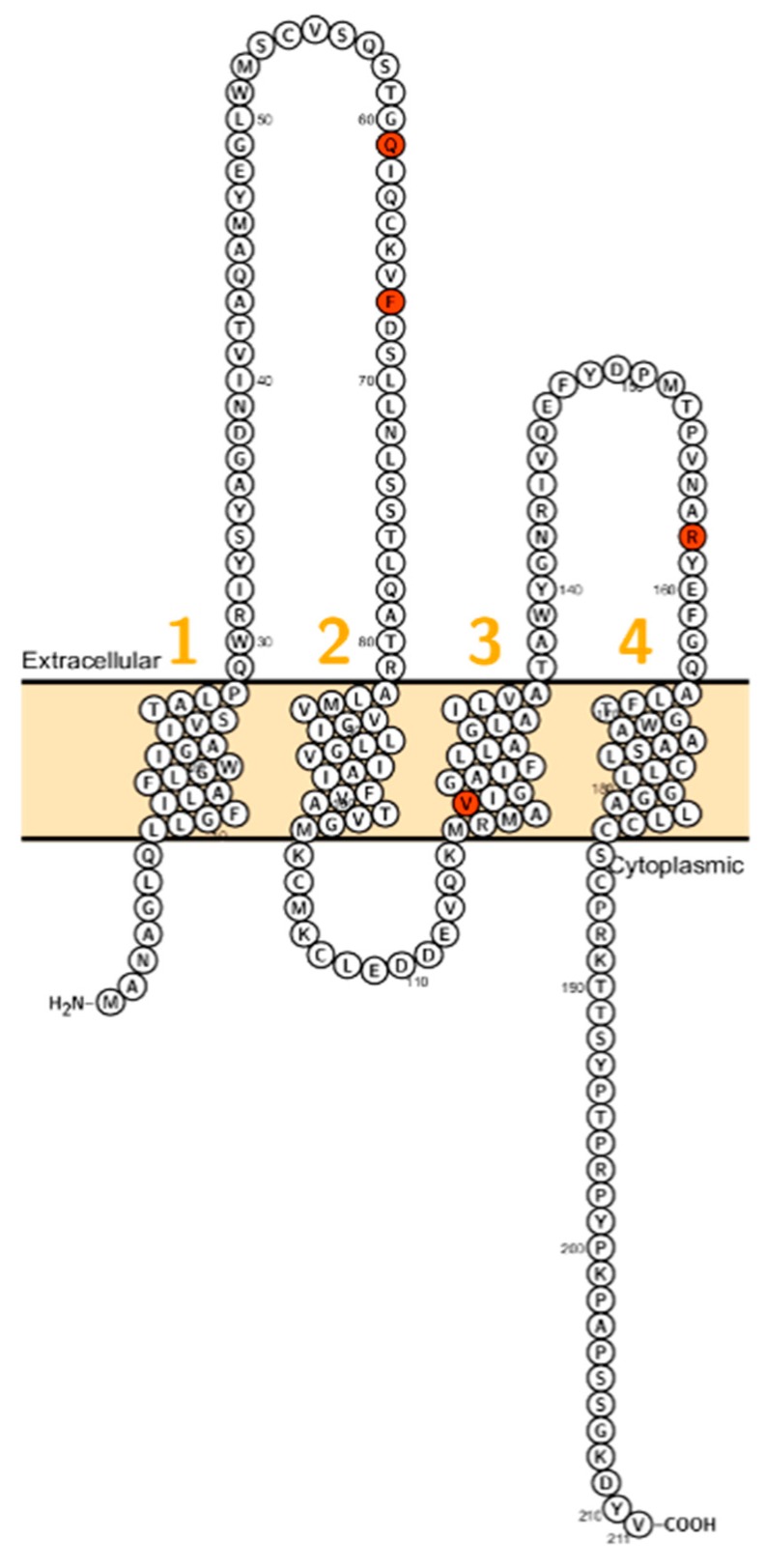
Topology of *CLDN1*. FASTA sequence of *CLDN1* (Uniprot accession no: O95832; plotted by Protter software http://wlab.ethz.ch/protter/). Each amino acid is shown as a single letter code and numbers (orange) indicate transmembrane domains. Mutations shown to be involved in human diseases are shown in red.

*CLDN1* consists of four exons encoding a 211 amino acid protein that conveys barrier properties [16]. It has been shown that claudin-1 interacts with claudin-3 and claudin-5, which are also barrier–forming claudins [32,33]. In the skin, *CLDN1* is expressed in the stratum corneum, stratum basale, stratum granulosum and stratum spinosum [34]. Further, it is expressed in the kidney [35], gall bladder [36], human ovarian epithelium [37] and the inner ear [38]. The expression of *CLDN1* is increased in colorectal cancer [39], lung carcinoma [40], cervical cancer [41] and reduced in larynx cancer [42]. Evans et al. showed that *CLDN1* is involved in Hepatitis C Virus entry into intestinal cells, which is presumably depending on the first extracellular loop (ECL, Figure 1) [43].

In 2002, Baala et al. described a family with autosomal dominant ichthyosis, alopecia, leucocytic vacuoles and sclerosing cholangitis (ILVASC; or neonatal ichthyosis with sclerosing cholangitis (NISCH-Syndrome OMIM 607626) assigned to chromosome 3q27–28 [44]. In 2004 Hadj-Rabia et al. identified in the same kindred a frameshift mutation in *CLDN1* leading to a premature stop codon at position 67 [18] (Figure 1). This finding, that ILVASC/NISCH syndrome is caused by mutations in *CLDN1* has been later confirmed by others [18,44,45,46]. In 2006, a neonate patient with erythroderma, massive lamellar desquamation and alopecia were reported. The hepatologic aspects were icterus, hyperbilirubinemia and increased biliary acids, liver biopsy showed panlobular cholestasis with acute hepatitis. All reported cases showed that *CLDN1* mutations in humans were not lethal and did not affect fertility.

Furuse and colleagues generated *Cldn1* deficient mice, which exhibited low body weight and died within the first day after birth, possibly attributable to an excessive trans-epithelial water loss (TEWL). *Cldn1* deficient mice displayed also altered lipid composition and defects of the stratum corneum of the skin. On the other hand, *CLDN1* deficient patients also displayed mild wrinkled skin and hyperproliferation of keratinocytes. However, only some patients had liver cell injury [18]. Whether knockout mice were affected by hepatic abnormalities has not been reported, probably due to the perinatal death.

Since the mouse model was limited because of its early lethality, a KD mouse approach was used, that showed reduced expression levels of *Cldn1* and *Cldn1* levels were associated with the severity of the phenotype [47]. *Cldn1* KD mice were born with wrinkled skin similar to KO mice, however, morphological examinations at 8 weeks of age revealed a normal development, which might be explained by the low but still expressed *Cldn1.* The underlying mechanism of TEWL was further investigated by Hirano et al. by using tamoxifen-induced epidermis-specific deletion of *Cldn1* in adult mice. Four days after induction, claudin-1 began depleting from basal layers and was undetectable in granular layers at day eight and tight junction leakage was observed. Neither TEWL nor stratum corneum defects were observed until day 18 suggesting that TJ deterioration is a prerequisite for stratum corneum defects [48].

### 2.2. Claudin 2

*Claudin-2* is one of the two claudins that were initially identified by Furuse et al. in 1998 [49]. In humans, its gene contains two exons encoding a 203 amino acid protein that constitutes cation- selective pores [50]. It is expressed in rat brain [51], proximal tubules of the kidney [52] liver, and epididymis [53].

Its expression is increased in colorectal cancer [54], active ulcerative colitis [55], a severe form of the coeliac disease [56] and inflammatory bowel disease [57] whereas it is downregulated in breast tumors and osteosarcoma [58,59]. It has been shown that miR-16 modulates *CLDN2* expression and causes dysfunctional barrier properties in inflammatory bowel disease [60].

*Cldn2* KO mice have been generated by Muto et al. [61]. Mice were morphologically normal at birth. The authors did not observe renal morphological abnormalities under both light and electron microscope. However, further analysis in proximal tubules revealed reduced absorption of Na^+^ and higher urinary fractional excretion of Ca^2+^. The same mice were investigated by a different group, focusing on small intestine showing that *Cldn2* deficient mice have slightly larger intestine and longer intestinal villi. They further demonstrated *Cldn2* dependent Na^+^ selective intestinal paracellular permeability. Matsumoto et al. focused on liver and biliary tissues of *Cldn2* deficient mice and similar to the kidney, no obvious morphological abnormalities were observed [62]. The detailed physiological analysis revealed a decreased bile flow in *Cldn2* deficient mice and four weeks under a lithogenic diet, KO mice developed gallstones as a consequence of altered bile composition and flow rate [62]. As *Cldn2* and *Cldn15* play an important role in paracellular ion flow, Wada et al. generated *Cldn2^-/-^ Cldn15^-/-^* double KO (dKO) mice [63]. Deficiency of both genes caused early death by week three, which was attributed to overt hypoglycemia of the dKO animals presumably caused by a disrupted Na^+^ flow, which is important for intestinal glucose absorption. Transgenic mice with colon specific overexpression of *Cldn2* displayed an enlarged colon and RNAs of genes involved in cell proliferation were found to be increased. In contrast, for inflammation related RNAs, the opposite was shown, in line with the fact that mice were protected against experimentally induced colitis [64].

**Figure 2:**
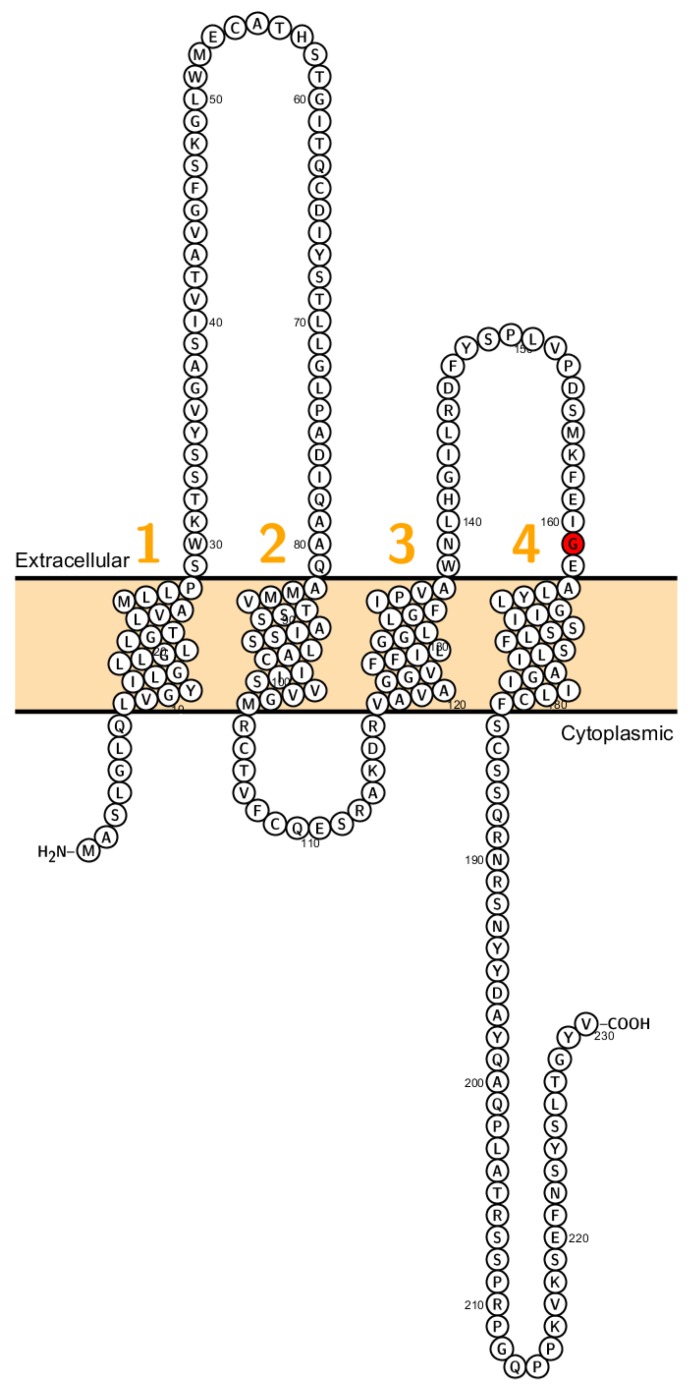
Topology of *CLDN2*. FASTA sequence of *CLDN2* (Uniprot accession no: P57739). Each amino acid is shown as a single letter code and numbers (orange) indicate transmembrane domains. Mutations shown to be involved in human diseases are shown in red.

Recently a missense mutation of Cldn2 associated with obstructive azoospermia in a four generation spanning family has been identified (Figure 2). Further, it has been shown that different claudins (*CLDN1, CLDN2, CLDN3, CLDN4* and *CLDN7)* are expressed in human epididymal tight junctions [65]. Except for *CLDN2* all these claudins cause, when eliminated, a disruption of the epithelial barrier. Therefore, it has been assumed that malfunction of these claudins might cause infertility in men but has not been proven so far.

### 2.3. Claudin 9

The Claudin-9 gene consists of a single exon encoding a 217 amino acid protein. It constitutes a barrier to K^+^ and Na^+^. Its expression was found in the inner ear, the liver [66] and the kidney [67].

Zhang et al. showed that claudin-9, together with claudin-6 mediates Hepatitis C Virus (HCV) entry into hepatoma cell lines [66]. Fofana et al. generated monoclonal antibodies against *CLDN9* which inhibit HCVpp (HCV pseudo particles) entry to *CLDN9* expressing cell lines [68].

The expression of *CLDN9* is increased in gastric cancer [69] and promotes cell proliferation and migration of lung metastasis [70]. In cervical carcinoma, RNA expression levels were found to be decreased [71].

**Figure 3:**
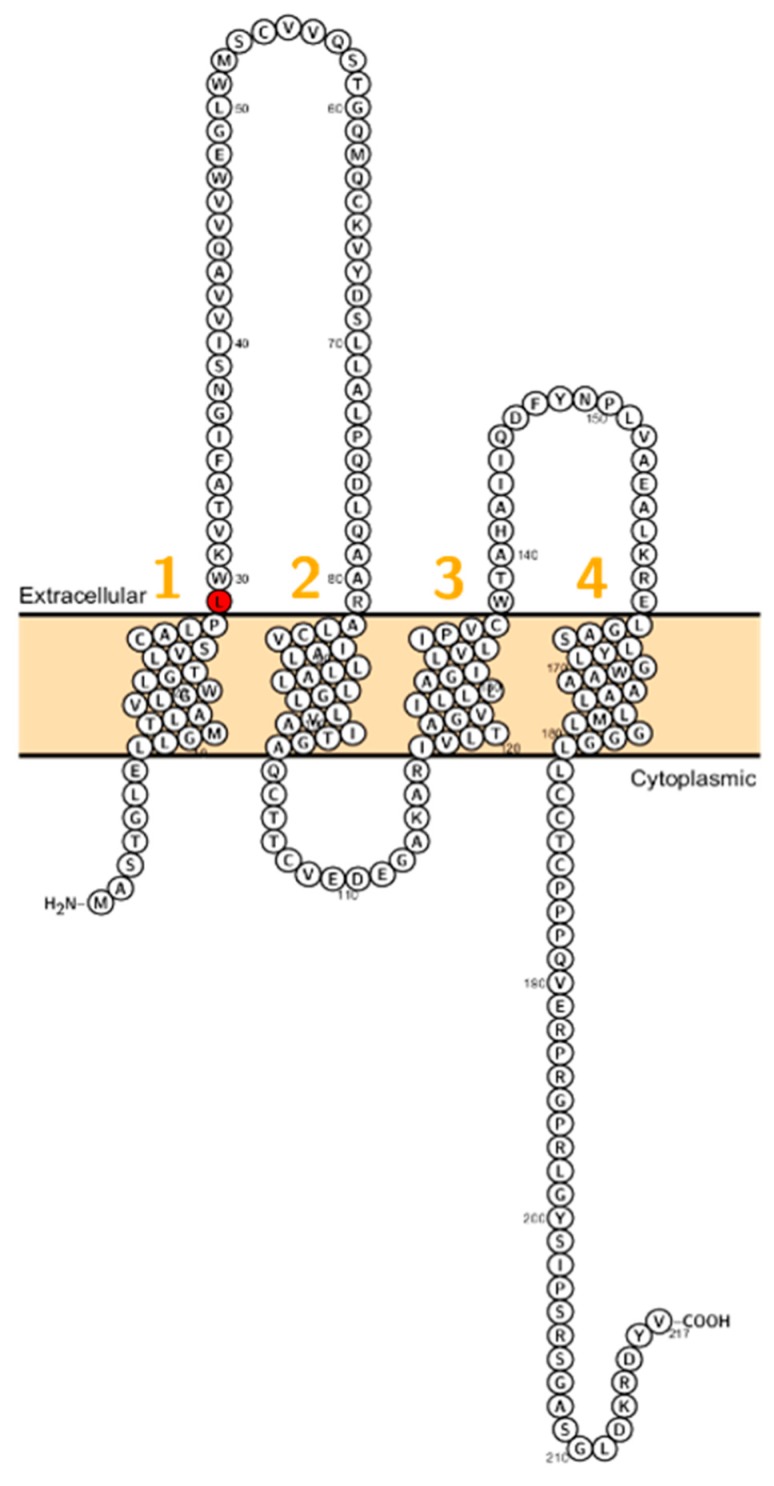
Topology of *CLDN9*. FASTA sequence of *CLDN9* (Uniprot accession no: O95484). Each amino acid is shown as a single letter code and numbers (orange) indicate transmembrane domains. Mutations shown to be involved in human diseases are shown in red.

Sineni et al. described patients with inherited autosomal recessive hearing loss who had a truncated variant of claudin-9 (p.L29fs). The mutation is located at the beginning of the first ECL (Figure 3). The truncated protein was not detected at the plasma membrane, indicating a dysfunctional TJ and thereby affecting the peri- and endolymphatic ion composition in the inner ear as the cause of hearing impairment [19]. Patients did not display coordination disturbances. This pathophysiological principle has been demonstrated in the past when mutations in BSND have been identified in patients with Bartter syndrome Type 4 [72].

Mice that carry a missense mutation in *Cldn9* were shown to be deaf. Further analysis showed that Reissner’s membrane was morphologically normal when compared to wild type mice and that only the basal but not the apical part of the cochlea was morphologically affected. Inner ear development was normal until postnatal day 14 (P14) then degeneration of the organ of Corti was observed. Since claudin-9 acts as a barrier for Na^+^ and K^+^, heterologous expression of the mutant in MDCK cells did not affect membrane targeting, indicating that this protein was not functional even when properly targeted and inserted into the TJ. In both, patients and mice hearing loss are age dependent. In mice at P14, outer hair cells (OHC) appeared normal whereas severe degeneration of OHC was observed at P80. In patients, a younger affected sibling had moderate hearing loss when compared to the older sibling. Neither mutant mice nor human patients had balance problems. Although *Cldn9* is expressed in various tissues, the authors did not report any abnormalities of other organs (e.g., kidney) yet.

### 2.4. Claudin 10

*CLDN10* consists of five exons encoding a 228 amino acid protein. Initially, there were two isoforms described [73], later Günzel et al. described four different splice variants mostly localized at TJs except for the ones that lack exon 4 [74]. Claudin-10a constitutes a pore for anions whereas claudin-10b forms a pore for cations [16,73]. *CLDN10b* is expressed in many tissues including the brain, lung [75], salivary gland [76], mammary gland [77] but *CLDN10a* is exclusively expressed in the kidney [73]. There are no isoform-specific antibodies, but according to RNA hybridization data, *Cldn10b* is highly expressed in medulla and *Cldn10a* in the cortex [73] and based on RNA-Seq data, *Cldn10a* is expressed in the proximal tubule (PT) and *Cldn10b* in thick ascending limb (TAL) [11].

Both isoforms interact with claudin-18 and claudin-19 in a yeast two-hybrid analysis [78], whereas immunofluorescence experiments on kidney sections did not demonstrate colocalization [79]. Additionally, claudin-10 has been implicated in left-right-patterning as well as in tumor progression and invasiveness [80].

**Figure 4:**
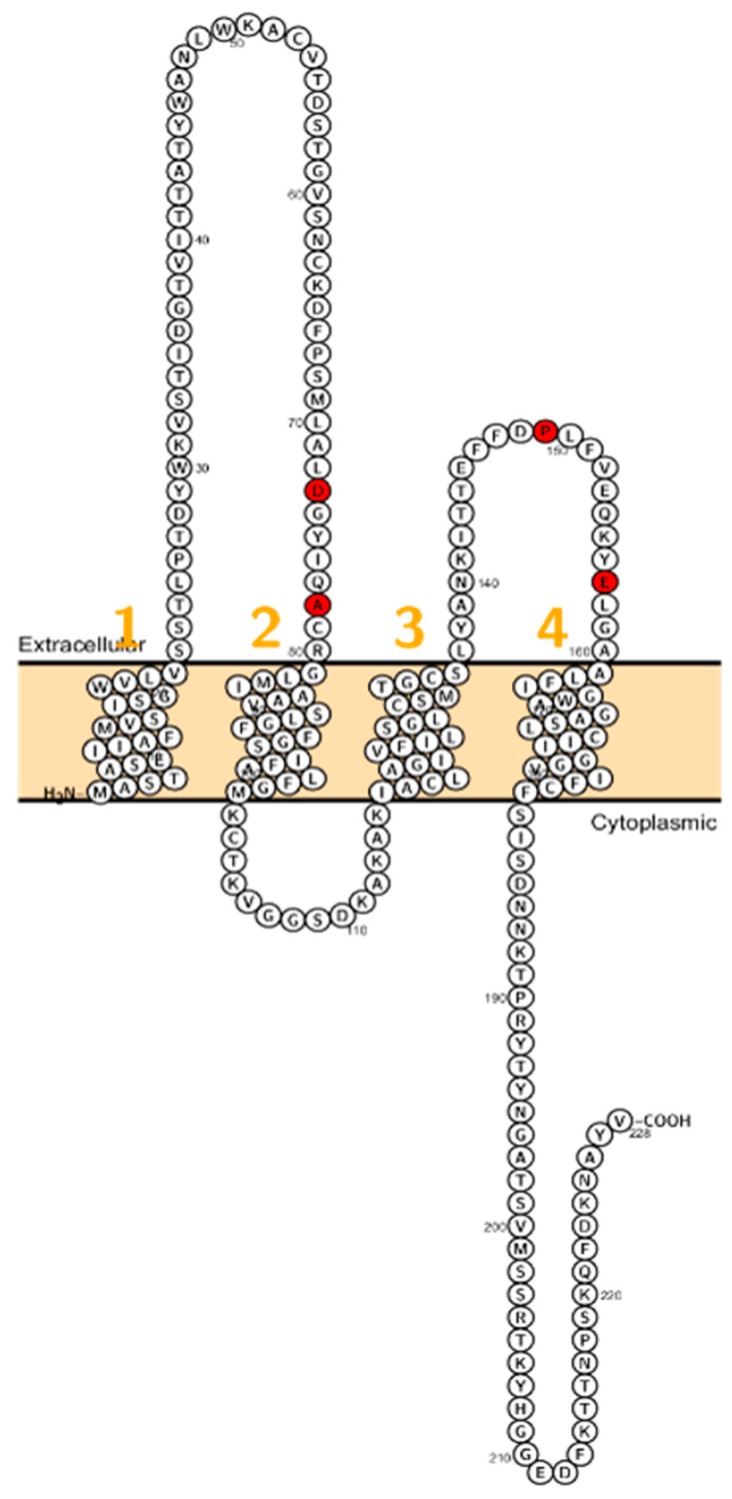
Topology of *CLDN10*. FASTA sequence of *CLDN10* (Uniprot accession no: P78369). Each amino acid is shown as a single letter code and numbers (orange) indicate transmembrane domains. Mutations shown to be involved in human diseases are shown in red.

Patients with a homozygous mutation in *CLDN10B* (N48K) showed anhidrosis, alacrima, dry mouth, and kidney failure with hypermagnesemia, low urinary Mg^2+^ and Ca^2+^ [81] (Figure 4). Patients did not display overt hypokalemia indicating only a mild renal electrolyte wasting although Meyers et al. described a patient with *CLDN10* mutation who initially presented with hypokalemia and follow-up examinations revealed a developing hypermagnesemia [82]. In fact, *CLDN10* patients exhibit a considerable range of hypohidrosis, hypolacrymia, ichthyosis and xerostomia and a decreased amount of saliva. Functional testing revealed that patients had decreased NaCl absorption in the TAL, too [82].

TAL specific knockout of both isoforms in mice resulted in polyuria, polydipsia and hypermagnesemia. Moreover, acidic urine and calcium deposits in the kidney (nephrocalcinosis) were observed. Quantitative expression analysis of TAL tubules revealed that expression of *Cldn10*, *Cldn16* and *Cldn19* was increased in kidneys of mutant animals [11].

Mice lacking claudin-16 and claudin-10 in the kidney were found to have normal Mg^2+^ in serum and absence of nephrocalcinosis. The authors demonstrated that the deletion of *Cldn10* and the loss of *Cldn16* led to an expansion of the DCT, especially which eventually resulted in increased resorption of Mg^2+^ [83].

### 2.5. Claudin 14

*CLDN14* is located on human chromosome 21 and consists of two exons encoding a 239 amino acid protein. It has five protein-coding transcript variants and is classified as a barrier forming claudin [15].

Overexpression of *CLDN14* is associated with gastric and hepatocellular forms of cancer [84].

*Cldn14* expression was found in outer and inner ear hair cells and also in the TAL, the DCT [85] and in the liver [12].

**Figure 5:**
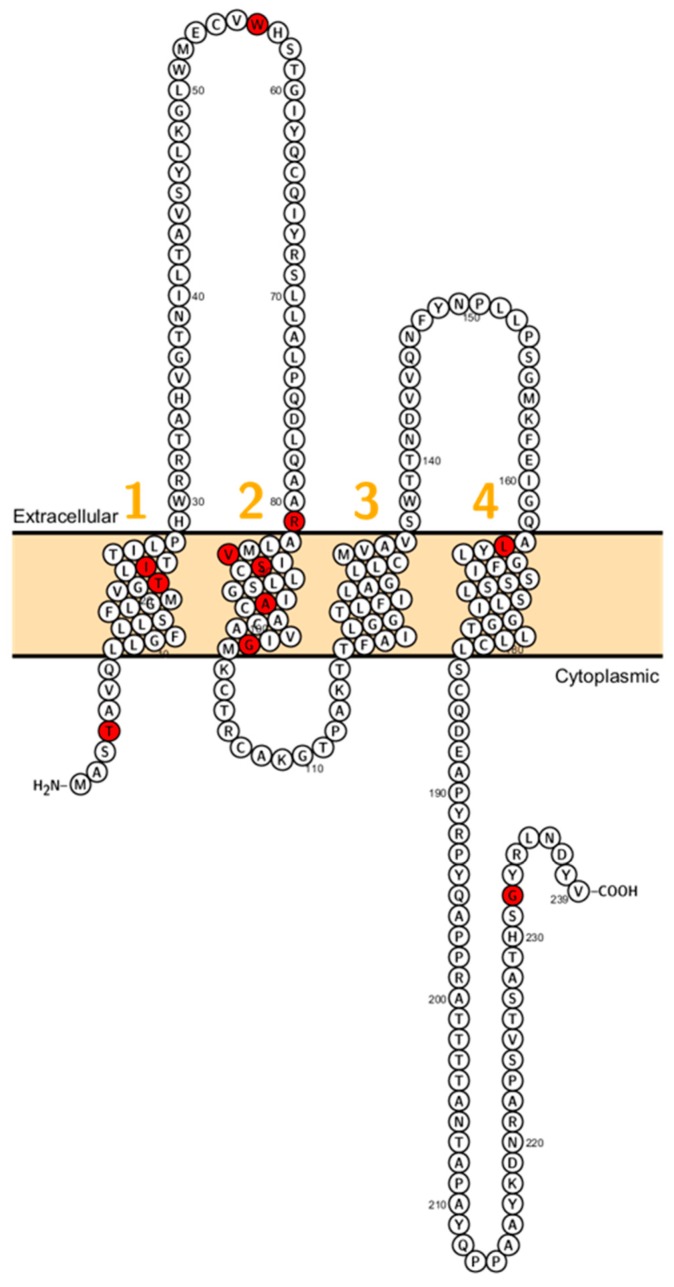
Topology of *CLDN14*. FASTA sequence of *CLDN14* (Uniprot accession no: O95500). Each amino acid is shown as a single letter code and numbers (orange) indicate transmembrane domains. Mutations shown to be involved in human diseases are shown in red.

Wilcox et al. reported on deaf patients from two large consanguineous families revealing a mutation (V85D) in *CLDN14.* This mutation is predicted to interfere with a phosphorylation site and this residue is conserved between *CLDN3* and *CLDN9* which are expressed in the inner ear too [86]. Immunocytochemistry experiments showed that the mutant failed to localize on the membrane [87]. After this initial study, several other mutations (W56, R81H, G232R) have been reported (Figure 5).

Examination of *Cldn14* mice that included kidney and liver along with 41 other tissues revealed no morphological differences between knockout and wild type animals. Inner hair cells and outer hair cells (OHC) seemed to develop normally. However, the rapid loss of OHC characterized by disorganization and loss of stereocilia was observed after the first week. Auditory brainstem response (ABR), tests revealed that knockout mice suffer from hearing loss by the third week of age [12].

Although initially no kidney abnormalities were observed neither in mice nor in patients, upon a high calcium diet, *Cldn14* deficient mice developed hypomagnesemia and hypocalciuria. Moreover, it has been shown that two microRNA regulate extracellular Ca^2+^ levels through suppressing *Cldn14* expression under normal diet conditions [85].

Transgenic overexpression of *Cldn14* in TAL of kidney resulted in hypercalciuria and hypermagnesuria [88].

In humans, the hearing loss occurred at the prelingual stage and the severity of hearing loss depends on the mutation. A patient with non-common mutation (A163V) was reported with hearing ability until the age of three, in line with the observation in mice [89].

### 2.6. Claudin 16

Claudin-16 is a pore forming claudin encoded by *CLDN16* gene [16]. It is expressed in duodenum, jejunum, ileum, colon [90], in tooth germ [91], in salivary glands [92], and also has been found in the endolymphatic duct of the inner ear [93]. The by far highest expression has been found in the thick ascending limb (TAL) of the kidney [94].

Several studies related overexpression of *CLDN16* with different cancer types including ovarian cancer [95] and papillary thyroid carcinoma [96]. Also, claudin-16 might play a role in cell proliferation and differentiation, e.g., breast cancer [97,98].

In the kidney, Claudin-16 is crucial for TJ-specific ion transport in the TAL and handles approximately 25% Ca^2+^ and 70% of Mg^2+^ reabsorption [99]. However, the exact role of claudin-16 remains a matter of debate. Hou and colleagues showed that claudin-16 enhances the permeability of monovalent cations, including Na^+^, than that of divalent cations, as Mg^2+^ (<50%) [90]. When *Cldn16* was deleted in mice, a cation-to-anion selectivity (P_Na_/P_Cl_) but no divalent-to-monovalent cation selectivity (P_Mg_/P_Na_) was observed [100]. Other groups have reported a selectivity of Claudin-16 for divalent cations Mg^2+^ and Ca^2+^ [101,102]. Hou et al. have described an interaction with claudin-19 as being necessary for correct TJ integration [78]. Additionally, it has been reported that claudin-14 reduces the cation permeability of claudin-16, but not for the claudin-19 in transfected LLC-PK1 cells. Based on this hypothesis, claudin-14 might act as a negative regulator of divalent cation permeation [85]. Split-ubiquitin yeast 2-hybrid (Y2H) membrane protein interaction assay showed that claudin-14 interacts with the claudin-16 [79]. Other known interactions reported are with syntaxin-8 [102] and with PDZ domain containing RING finger 1 (encoded by *PDZRN*) [103] by electrophysiological experiments [104].

**Figure 6:**
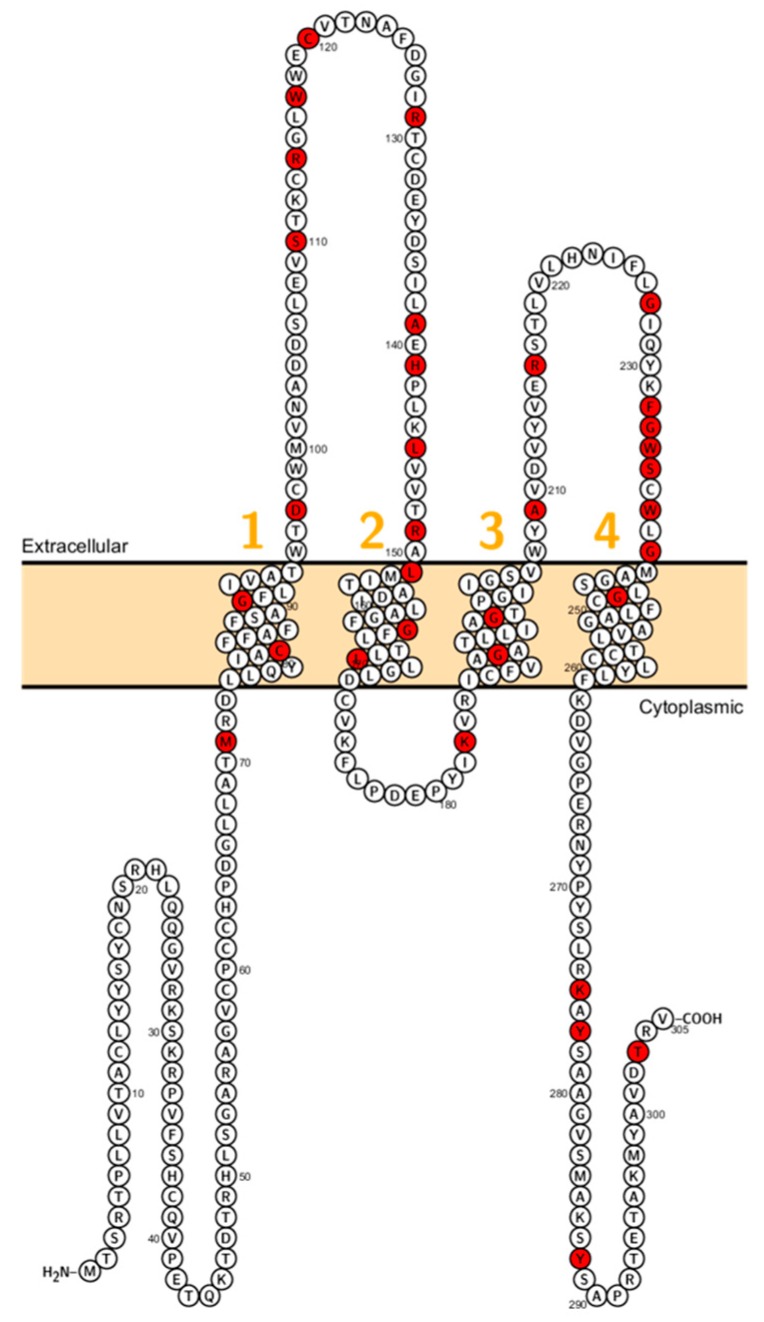
Topology of *CLDN16.* FASTA sequence of *CLDN16* (Uniprot accession no: Q9Y517). Each amino acid is shown as a single letter code and numbers (orange) indicate transmembrane domains. Mutations shown to be involved in human diseases are shown in red.

Mutations in human *CLDN16* cause an autosomal recessive disorder called *Familial hypomagnesemia with hypercalcinuria and nephrocalcinosis* (FHHNC) [105] (Figure 6). Other phenotypic features are incomplete distal tubular acidosis, impaired bone homeostasis [94], hypocitraturia and hyperuricemia which can be considered a secondary effect of renal insufficiency. To date, about 66 mutations including missense/nonsense, splicing, small deletion and small indels have been identified. Although still a matter of debate, it is believed that claudin-16 and claudin-19 form a heterodimer/tetramer essential for the divalent cation selectivity of the paracellular channels at the TAL and it has been demonstrated that some mutations disrupt this interaction [106].

*Cldn16* deficient mice exhibited hypomagnesia and hypercalciuria and a low urinary pH but did not show nephrocalcinosis which could be explained by the upregulation of several genes (e.g., *Trpm6, Trpv5, Cnnm2*) involved in calcium and magnesium transport or the altered pH. Moreover, mice did not show renal insufficiency [13].

Besides, knockdown mice were generated by RNA interference technology [100] which phenocopied the main features of FHHNC including hypercalcinuria, hypomagnesemia, nephrocalcinosis and urinary Ca^2+^ and Mg^2+^ wasting without showing nephrocalcinosis or renal insufficiency. *Cldn16* KD animals showed increase of 1,25-dihydroxychlecalciferol [100].

For Cldn16 Patients, a genotype-phenotype correlation has been reported [94]. In sharp contrast to humans, although mice recapitulate many features of FHHNC like hypomagnesemia and hypercalciuria, they do not display nephrocalcinosis nor renal insufficiency [100]. Most of the patients with FHHNC nephrocalcinosis develop end stage renal disease with a need of renal transplantation [107]. Recently, amelogenesis imperfecta has been related to the absence of Claudin-16 in the ameloblasts in humans and mice [105] as an additional role of Cldn16 deficiency.

### 2.7. Claudin 19

The CLDN-19 gene encodes a 224 amino acid protein. Claudin-19 is a barrier forming claudin and is expressed in the thick ascending limb of the kidney and the retinal pigment epithelium (RPE) and the sheath of myelinated peripheral nerves. Additionally its expression was reported detected in the inner ear [38], stomach [108] and lung [109]. Like claudin-16, claudin-19 plays a major role in the permeability and selectivity of the TJ in TAL.

Claudin-19 interacts with claudin-16, both are involved in the reabsorption of divalent cations in the TAL [79,110]. Gong et al. isolated a stable dimer of claudin-16 with claudin-19 from transfected HEK293 cells and Sf9 cells [85]. The dimerization occurs through the cis-association of the third and the fourth transmembrane domain in both proteins [111]. This hypothesis is complemented by in vivo transgenic animal models, deletion of claudin-16 rendered claudin-19 delocalization from the TJ and vice versa [78]. Selective mutations disrupt the dimerization, triggering a loss of the transport function with FHHNC disease is the consequence [112]. 

**Figure 7:**
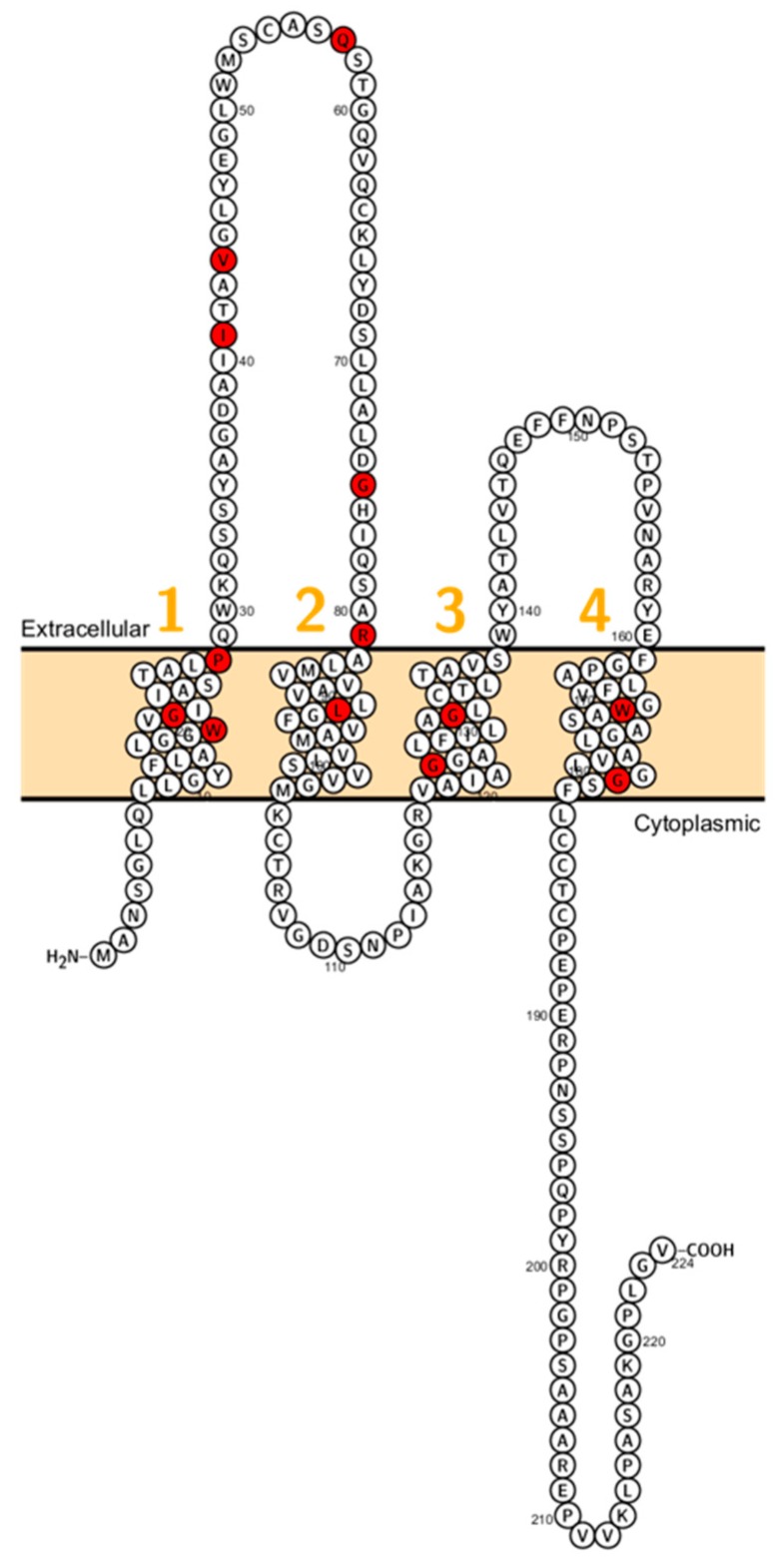
Topology of *CLDN19*. FASTA sequence of *CLDN19* (Uniprot accession no: Q8N6F1). Each amino acid is shown as a single letter code and numbers (orange) indicate transmembrane domains. Mutations shown to be involved in human diseases are shown in red.

Moreover, claudin-19 and ZO-1 are found by co-immunoprecipitation forming a complex in Madin-Darby canine kidney (MDCK) cells [97]. Meier et al. reported patients presenting by a phenotype of FHHNC disease but with the ocular disease [113,114]. All individuals of these families suffered from severe visual impairment, characterized by macular colobomata, significant myopia, and horizontal nystagmus. The genotype of these patients did not show a mutation in *CLDN16*, but Konrad and colleagues identified three mutations (G20D, Q57E) in *CLDN19*, recapitulating CLDN16 defects but, ocular defects, too. Other reported mutations (L90P and G123R) disrupt the interaction between claudin-16 and claudin-19 [23,112] (Figure 7).

*Cldn19* KD mice showed a reduction of Mg^2+^ in plasma and excessive losses of Ca^2+^ and Mg^2+^ levels in kidney similar to *Cldn16* KO mice [78]. These mutations are the cause of FHHNC diseases, in which interaction between claudin-16 and-19 is disrupted. The dissociation of these proteins can trigger a loss of the transport function of them and cause FHHNC [112]. On the other hand, knockout mouse of *Cldn19* provokes renal reabsorption deficiency [79].

## 3. Discussion

Twenty years ago, the seminal work of Lifton’s group described mutations in a gene coding for a TJ protein (initially named paracellin-1 and thereafter classified as claudin-16) as being causative for a human disorder (FHHNC) [99]. *CLDN16*, being the first claudin of a continuously expanding group of claudins causing human disorders, has been subject to various investigations on expression, function and also on possibilities of pharmaceutical interventions [115]. To gain further insights, mouse models, either as knockdown or knock out have been established (Table 1). Such models recapitulated some (Hypercalciuria, Hypomagnesemia) but not all (Nephrocalcinosis and renal insufficiency) of the hallmarks of human FHHNC, demonstrating the benefits but also the limitations of such models of TJ disorders. Recently the association of claudin-16 and amelogenesis imperfecta in men and mice has been shown [91].

As the example of claudin-16 demonstrates, disorders that are caused by mutations in the corresponding claudin genes are not restricted to a given organ but rather should be considered as ‘claudinopathies’. This view would not restrict the consequences to tissue-specific local or regional expression but appreciates the function and loss in all of the organs and tissues where a given claudin is expressed. Moreover, in a given organ, multiple different claudins are expressed and can cause, when mutated a similar phenotype albeit having different underlying pathophysiology. For example, kidney stones are a worldwide problem that affects 12% of the world population regardless of gender [116,117]. Besides *CLDN16* and *CLDN19*, in 2009, a genome-wide association study on an Icelandic and Dutch population revealed that a *CLDN14* variant (rs219780[C]) is associated with kidney stones too.

In this perspective, animal models, especially mice with all their limitations, are indispensable to study the consequences of claudin deficiency.

## Figures and Tables

**Table 1:** Overview of claudins causing human diseases and corresponding mouse models. The existence (by publication) is indicated by (+) while (−) indicates the absence of a corresponding model.

	Human		Mouse			
Claudin	Human Disorder	Hallmarks	Knockout	Knockdown	Conditional Knockout	Transgenic Overexpression
1	NISCH Syndrome	Ichthyosis	Ichthyosis	Ichthyosis	+	+
2	Obstructive azoospermia	Male infertility	(*Cldn2*^−/−^) Larger intestine (*Cldn2^−/−^ Cldn15^−/−^*) Hypoglycemia Perinatal lethality	−	−	Larger colon
9	Nonsyndromic sensorioneural deafness	Deafness	Deafness	−	−	−
10	HELIX syndrome	Hypokalemia Hypermagnesemia Nephrocalcinosis	Perinatal lethality	−	Hypokalemi Hypermagnesemia Nephrocalcinosis	−
14	Nonsyndromic sensorioneural deafness	Deafness	Deafness	−	−	Hypercalcuria Hypermagnesemia
16	FHHNC	Hypercalciuria Renal insufficiency Hypomagnesemia Nephrocalcinosis	Hypercalciuria Hypomagnesemia	Hypercalciuria Hypomagnesemia	−	−
19	FHHNC + Eye Involvement	Hypercalciuria Renal insufficiency Hypomagnesemia Nephrocalcinosis Eye involvement	Hypercalciuria Hypomagnesemia	Hypercalciuria Hypomagnesemia Eye abnormalities	−	−
